# Phylogenetic analysis of congenital rubella virus from Indonesia: a case report

**DOI:** 10.1186/s12887-022-03775-4

**Published:** 2022-12-13

**Authors:** Elisabeth Siti Herini, Agung Triono, Kristy Iskandar, Albaaza Nuady, Lucia Hetty Pujiastuti, Marcellus, Andika Priamas Nugrahanto, Musthofa Kamal, Gunadi

**Affiliations:** 1 Department of Child Health, Faculty of Medicine, Public Health and Nursing, Universitas Gadjah Mada/Dr. Sardjito Hospital, Yogyakarta, 55281 Indonesia; 2 Department of Child Health/Genetics Working Group, Faculty of Medicine, Public Health and Nursing, Universitas Gadjah Mada/UGM Academic Hospital, Yogyakarta, 55281 Indonesia; 3 Department of Ophthalmology, Faculty of Medicine, Public Health and Nursing, Universitas Gadjah Mada/Dr. Sardjito Hospital, Yogyakarta, 55281 Indonesia; 4 Department of Ophthalmology, Dr. Soeradji Tirtonegoro Hospital, Klaten, 57424 Indonesia; 5 Pediatric Surgery Division, Department of Surgery/Genetics Working Group, Faculty of Medicine, Public Health and Nursing, Universitas Gadjah Mada/Dr. Sardjito Hospital, Yogyakarta, 55281 Indonesia; 6 World Health Organization (WHO) Indonesia Representative, Jakarta, 12940 Indonesia

**Keywords:** Congenital rubella syndrome, Eye lens, Genotype, Indonesia, Phylogenetic analysis

## Abstract

**Background:**

Rubella is a common inherited infection resulting in congenital cataracts and a significant cause of permanent vision loss in developing countries. In 2016, Indonesia had the highest number of congenital rubella syndrome (CRS) cases globally. Here, we report the first genotype of the rubella virus extracted from the eye lens from a child with congenital cataracts due to CRS.

**Case presentation:**

A female neonate was delivered by an elective caesarean delivery with normal birth weight at term from a 24-year-old mother in the rural setting. The baby presented with bilateral congenital cataracts, small-moderate secundum atrial septal defect, severe supravalvular pulmonary stenosis, and profound bilateral hearing loss. She also had microcephaly and splenomegaly. The patient's serology showed persistent positive IgG for rubella virus at the age of four years and four months. Following extraction during cataract surgery, viral detection of the lenses identified the presence of rubella. Phylogenetic analysis confirmed that the virus was grouped into genotype 1E.

**Conclusions:**

Our study reports the first phylogenetic analysis of the rubella virus extracted from the eye lens of a child with CRS in Indonesia. The detection of the rubella virus from eye lenses is remarkably promising. Our findings also emphasize the importance of molecular epidemiology in tracking the origin of rubella infection toward achieving virus eradication.

**Supplementary Information:**

The online version contains supplementary material available at 10.1186/s12887-022-03775-4.

## Introduction

Rubella or German measles is caused by the rubella virus that belongs to the family of Matonaviridae and is the only member of the genus Rubivirus [1]. Its genome consists of 9,762 nucleotides which encode three structural proteins (C, E1, and E2) and two non-structural proteins (p90 and p150) [2]. The World Health Organization (WHO) recommends using a region of 739 nucleotides (nt) (nt 8731 to 9469) within the E1 gene for routine molecular characterization. Rubella viruses are classified into two clades according to these sequences of nucleotides [3]. Clade 1 consists of 9 recognized genotypes (1B, 1C, 1D, 1E, 1F, 1G, 1H, 1I, and 1 J) and 1 provisional genotype (1a) while clade 2 contains 3 accepted genotypes (2A, 2B, and 2C) [3, 4]. Only four genotypes (1E, 1G, 1 J, 2B) are now commonly detected and reported, with 1E and 2B being the most frequently identified with wide geographic distribution [5].

Rubella typically manifests as a mild disease with symptoms such as fever and skin rash. However, rubella infection during pregnancy, especially in the first trimester, results in congenital anomalies, prematurity, and fetal death. Infected newborns along with the anatomic defects constitute the congenital rubella syndrome (CRS) [6]. In 2010, the estimated incidence of CRS ranged from 90 to 121 cases per 100,000 live births, with the highest numbers predicted in Africa and Southeast Asia region [7]. The rubella vaccine was introduced as part of the national immunization program in 2017–2018 and has achieved national coverage of 87.33% in that period [8]. The CRS incidence was 0.39 per 1000 live births in pre-campaign and 0.08 in postcampaign [9]. The coverage of RV-containing vaccine in 2019, 2020, and 2021 for infant aged 9 months were 95.2%, 86.9%, and 87.3%, respectively [10]. COVID-19 pandemic contributed in the declining in the rubella vaccination coverage.

Genotype analyses of the rubella virus are essential for achieving virus eradication. These data can be used to track virus control and elimination progress, help case classification, and trace transmission pathways [3]. Analyses of rubella strains in Indonesia have only been done to a minimal extent: only three sequences of the rubella virus genotype 1E lineage have been deposited to the GenBank, which came from travelers returning to the United States and Japan from Indonesia [11]. Moreover, to our best knowledge, no studies have been conducted regarding virological surveillance of the rubella virus in Indonesia after the vaccination campaign. This paper aims to: (1) report the first genotype of the rubella virus extracted from the eye lens, which was found in a child with congenital cataracts due to congenital rubella infection; and (2) report the first finding of genetic CRS from Indonesia to the GenBank.

## Case presentation

### Clinical findings

A female neonate was born by an elective cesarean section due to cephalopelvic disproportion, with a normal birth weight (2,540 g) at term, to a 24-year-old secundigravida mother in the rural setting. Serological testing for TORCH infection for the mother was not done previously. Routine ultrasound screening was within the normal limit. The patient's mother was unaware of having any rubella symptoms nor did she have contact with any patient with rubella during pregnancy. Her mother also had never received the rubella vaccine. There was no history of consanguinity, no known inherited conditions, nor congenital abnormalities and no other ocular malformations occurring in the family.

On physical examination, the neonate had microcephaly and a non-dysmorphic face. Complete cardiac examinations showed a small-moderate secundum atrial septal defect and severe supravalvular pulmonary stenosis which were confirmed using echocardiography. Assessment for hearing impairment using the Brainstem Evoked Response Audiometry showed bilateral profound hearing loss. The abdominal examination identified splenomegaly. Unfortunately, the serology testing for diagnosis of congenital infection, including rubella, was not conducted in the first year of life due to the late case presentation and findings. Based on these clinical findings, she was categorized as clinically compatible CRS [12].

**Fig. 1:**
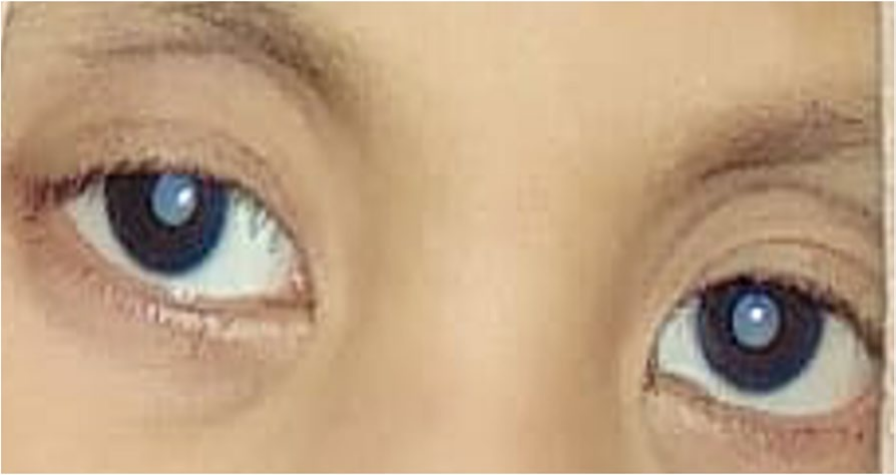
Bilateral cataract in four years and three months of age a patient with congenital rubella syndrome

At four years and three months old, due to visual impairment, the child was brought to the ophthalmologist for an eye examination (Fig. 1). The patient's serology results showed positive IgG for the rubella virus. Subsequently, she underwent cataract surgery at four years and four months old. Percutaneous transcatheter ballooning valvuloplasty was performed for the congenital cardiac problem at five years old. There were no complications after both surgeries. The visual acuity for both eyes is 1/60. The vision could not be optimal due to the infection resulting in retinopathy of the entire retinal area. There are no activity limitations after the surgery. The patient is still routinely checked every two months until the writing of this paper.

### Rubella virus genotype

**Fig. 2:**
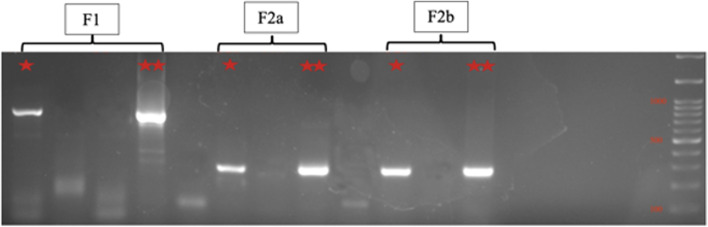
Nested-PCR electrophoresis showed F1 (727 nt) and F2 (271 nt) of the sample. **, positive control (rubella vaccine). Full-length gels are presented in Supplementary Fig. 1

Viral RNA was extracted from lens samples using the QiaAmp Viral RNA Mini Kit (Qiagen, Valencia, CA). Reverse transcription-polymerase chain reaction (RT-PCR) assay was used to detect the existence of rubella virus RNA in lens samples using the SensiFAST™ Probe Lo-ROX One-Step Kit (Bioline). The assay amplified a 185 nucleotides fragment of the E1 coding region. The nested RT-PCR assays were designed to amplify the RV E1 coding area segments, including the WHO-recommended 739nt region for rubella genotyping using the Center of Disease Control (CDC) protocol [13] (Fig. 2, Supp. Figure 1).

**Fig. 3:**
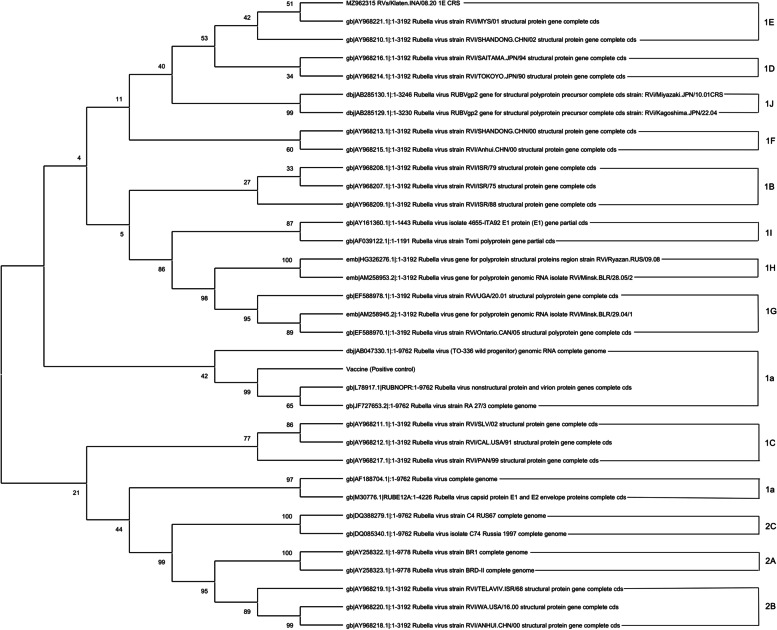
Phylogenetic analysis of the rubella virus strains that were found in this study belonged to genotype 1E

The E1 glycoprotein gene's 739nt window could be amplified, and the PCR products sequenced. Genotype RVs were determined bidirectionally using a BigDye® Terminator v3.1 Cycle Sequencing Kit and an Applied Biosystems PRISM 3730xl Genetic Analyzer. All analyses were conducted using CDC methods in the Molecular Evolutionary Genetics Analysis version 10 (MEGA X) program [14, 15]. Phylogenetic analysis with WHO reference viral genomes revealed that our sample belonged to genotype 1E (Fig. 3).

**Fig. 4:**
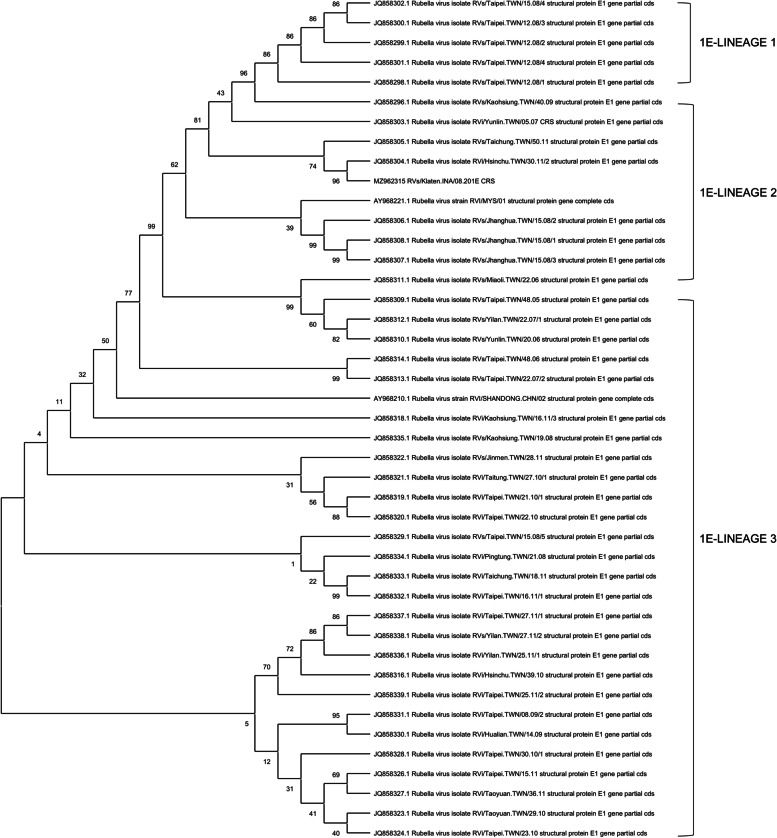
Phylogenetic analysis of genotype 1E to classify into the distinct lineage. Our sample was classified into L2

Subsequently, we performed a phylogenetic analysis using the maximum-likelihood method to classify genotype 1E into five distinct lineages (L0-L4). Based on the classification, our sample was grouped into L2 (Fig. 4). The nucleotide sequences from the sample, found to contain RV of genotype 1E, were submitted to the GenBank database and can be found under accession numbers MZ962315.

## Discussion and conclusions

Indonesia was once the country with the highest prevalence of rubella outbreaks in 2016 [16]. However, China and Japan are the only Asian nations that routinely provide virological data, with a mean of 40 and 30 viral sequences reported every year, respectively [3]. In contrast, only three sequences of the virus from Indonesia were registered in GenBank, from one patient who returned to the United States in 2011 (Hendersonville. NC.USA/15.11, accession no. JX477651 [1E]) and two patients who returned to Japan in 2017 (RVs/ Yokohama.JPN/3.17 [1E] and RVs/Osaka. JPN/41.17 [1E]) [11]. Identifying the rubella virus strain is essential in verifying the elimination of rubella and the dissolution of indigenous rubella virus strains. Because of the worldwide convergence of the presently circulating RV genotypes, it is challenging to differentiate imported strains from indigenous strains in any nation based only on genotyping data [17].

Our findings are the first report associated with congenital rubella genotyping following the measles-rubella vaccination campaign in Indonesia and the first report with cataract lens as the sample source. The campaign was launched in August–September 2017 in Java and August–September 2018 for other regions (outside Java). Before the campaign, Genotype 1E was the most prominent rubella genotype that circulated throughout Indonesia [18] and formed an outbreak in multiple countries in Asia [19]. However, this viral isolate has never been submitted to GenBank. To date, Indonesia has never officially reported the genotype of the rubella virus.

Our study reported one genotype of rubella which is 1E. In 2011, the same genotype was registered in GenBank as the first sequence from an Indonesian patient who returned to the United States [11]. Genotype 1E has been collected in ten countries, which are primarily located in Asia since 2010 [3]. In 2017, the rubella genotype was reported in a Japanese tourist who came to Indonesia 14 days before the onset of symptoms. The only symptoms were low-grade fever, sore throat, and rash that appeared on day four after onset. The genotype obtained was 1E, which is consistent with what we obtained in this study. He was infected in Indonesia because there has not been a domestic rubella epidemic found in Japan since 2013 [11]. Based on the lineage of genotype 1E, our finding is classified into L2, which is consistent with the genotype deposited before from Japanese and American tourists who visited Indonesia. This finding supports the premise that IE-L2 strains are indigenous strains that circulate throughout Indonesia. This case indicates that 1E is still circulating, although in a restricted geographical region [3, 19].

More than a million children in Asia are blind due to pediatric cataracts [20]. Rubella is a common inherited infection causing congenital cataracts and an important cause of lifelong visual deprivation in developing countries [21, 22]. Children with congenital cataracts can still acquire the best visual acuity if they undergo a well-defined treatment protocol regarding the time of surgery, postsurgical visual rehabilitation, with proper and prompt management of complications [22]. A five-year study conducted in Yogyakarta, Indonesia, found that 66.7% of laboratory-confirmed CRS cases had congenital cataracts [23]. The cataract condition may be unilateral or bilateral, and the most common morphologies were total, zonular, and membranous cataracts [22]. Unfortunately, the cataract case finding in this patient was very late because the patient came to the hospital with light perception in visual acuity. After the cataract surgery, the visual acuity was improved but could not be optimal due to the infection resulting in retinopathy of the entire retinal area. Early diagnosis and intervention can provide a better prognosis for children's vision.

Our findings also prove that the rubella virus can still be found in cataract lenses of patients with CRS even though rubella IgM was negative for the patient in the serology examination. It can also be found when the patient's age has already passed twelve months, even until almost five years. This finding indicates that the rubella virus can still be found within a few years after birth in lens material [24, 25]. In another study, congenital cataracts in patients with CRS were commonly found in younger patients aged < 12 months, while the oldest one was at 32 months old [25]. Comparing our findings to previous studies, it becomes more remarkable that the persistent rubella virus in the eye lens was still found in a four years and four months old female patient with CRS. The persistent rubella virus found in lenses in patients with CRS with ocular abnormalities is because the virus reaches the lens before the lens capsule develops, which would ordinarily function as a barrier to the virus [25].

The diagnosis of CRS in this study was made relatively late. Studies showed that most laboratory-confirmed CRS cases were diagnosed among cases who were 0- < 6 months of age which contradicts the late presentation of our patient [26–28]. In Indonesia, the diagnosis of CRS relies on serological testing, which is less sensitive [29]. Serological testing presents only indirect indicators of infection and may be misleading due to delayed antibody production and false-positive results. For CRS diagnosis, nested RT-PCR is a promising method with a more sensitive and rapid technique than the conventional method of virus isolation and serology [30]. However, in a developing country such as Indonesia, the health system is not equally accessed by all members of the society. In this low-resource setting, there are inequalities in access to health care due to the diversity in geographical areas of Indonesia, health insurance coverage, education levels, and economic background.

In all of the cases reported in this study, the mothers of the patients were unaware of the symptoms of rubella infection during pregnancy due to the mild symptoms of a rubella infection such as fever, rash, cough, and adenopathy, which are usually considered as common ailments. Therefore, the diagnosis of rubella infection in pregnancy is very difficult [31]. For developing countries such as Indonesia, with uncontrolled rubella infection rates, a higher vigilance is needed by: (1) serological testing for pregnant women with similar symptoms, (2) rubella vaccination in women of reproductive age before planning a pregnancy, and (3) increasing coverage of rubella vaccination in Indonesia.

The challenge of eliminating congenital rubella syndrome is also more significant than before the COVID-19 pandemic. Several immunization programs have declined due to parents' fear of coming to health service providers, including the hospital. The decline in the measles-rubella vaccination coverage is likely to increase their mortality and morbidity. The pandemic also slowed the implementation of measles and rubella mass vaccination campaigns in many regions and delayed various monitoring and evaluation activities. According to UNICEF and the WHO, many children in remote areas do not receive the first dose of a measles-containing vaccine as part of the routine immunization program each year [32]. As the national coverage in the scheduled COVID-19 vaccinations has increased, a more complete immunization program is slowly emerging and reestablishing the public health platforms needed to meet the goals of disease control and eradication [33].

Rubella is a common inherited infection that causes congenital cataracts and is a major cause of lifelong vision loss and poor quality of life in children and their families. Children born with congenital cataracts can still achieve the best visual acuity if they follow a well-defined treatment protocol that includes the timing of surgery, postoperative visual rehabilitation, with proper and prompt management of complications. Unfortunately, the cataract case in our patient was discovered very late after she presented to the hospital with light perception in visual acuity. Early detection and intervention can improve the prognosis for children's vision.

Our study presented the first genotype of the rubella virus extracted from the eye lens, which was discovered in a child with congenital cataracts caused by congenital rubella infection. Additionally, our findings demonstrate that the rubella virus can be detected in the cataract lenses of patients with CRS even when the patient tested negative for rubella IgM in the serology examination. It can also be detected when the patient is older than twelve months, and up to five years old. The persistence of the rubella virus in lenses of CRS patients with ocular abnormalities is due to the virus infecting the lens before the lens capsule develops, which normally serves as a barrier to the virus.

Based on the genotype 1E lineage classification, our discovery is classified as L2, which are indigenous strains that circulate throughout Indonesia. This case suggests that 1E is still circulating, albeit in a limited geographical region. The detection of rubella virus RNA using lens material is remarkably promising. This approach can help establish the diagnosis of CRS in older children. This information can be critical for ophthalmologists and pediatricians, especially in patients with unusual clinical signs.

Our findings also contribute to establishing baseline data of RV genotypes in Indonesia. These results emphasize the relevance of molecular epidemiology in tracking the origin of rubella outbreaks, such as in Indonesia, and the importance of generating enough sequencing data to be made available in GenBank. Furthermore, our data emphasize the importance of strengthening laboratory and epidemiologic surveillance of rubella, particularly in Indonesia, which is mandatory for rubella control and elimination.

## Supplementary Information

**Additional file 1: Supplementary Figure 1.** Representative electrophoresis result of nested-PCR electrophoresis.

## Data Availability

The datasets are publicly available at GenBank (https://ncbi.nlm.nih.gov/nuccore/MZ962314; Accession number: MZ962315).

## Abbreviations

CRS: Congenital rubella syndrome
RV: Rubella virus
WHO: World Health Organization
