# Supplementary figures and images for: Phylogenetic analysis of congenital rubella virus from Indonesia: a case report
Source: BMC Pediatr. 2022 Dec 13;22:713. doi: 10.1186/s12887-022-03775-4 (PMC9745697; doi:10.1186/s12887-022-03775-4)

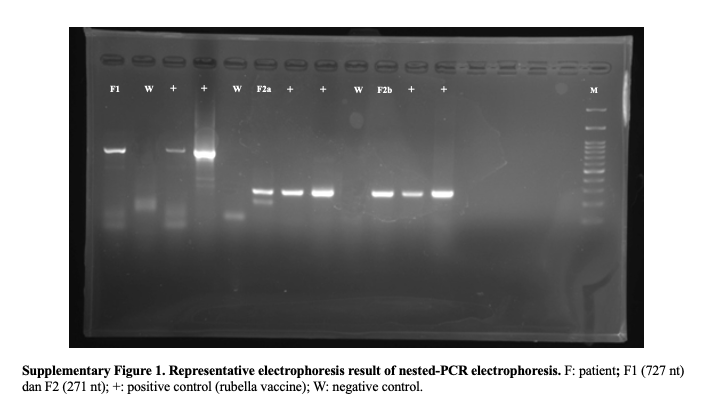

Supplement: Supplementary file 1 — Additional file 1: Supplementary Figure 1. Representative electrophoresis result of nested-PCR electrophoresis. [file 12887_2022_3775_MOESM1_ESM.tiff]
